# Chitosan-Enhanced pH-Sensitive Anthocyanin Indicator Film for the Accurate Monitoring of Mutton Freshness

**DOI:** 10.3390/polym16060849

**Published:** 2024-03-20

**Authors:** Yanli Ma, Lei Wen, Yaobo Liu, Pengfei Du, Peng Hu, Jianfang Cao, Weiting Wang

**Affiliations:** 1 Shandong Provincial Key Laboratory of Agro-Products Processing Technology, Key Laboratory of Novel Food Resources Processing, Ministry of Agriculture, Institute of Food & Nutrition Science and Technology, Shandong Academy of Agricultural Sciences, Jinan 250100, China; dr_mayanli@163.com (Y.M.);; 2 College of Life Sciences, Yantai University, Yantai 264005, China

**Keywords:** anthocyanin, chitosan, pH response sensitivity, indicator

## Abstract

Natural anthocyanin indicator films with an excellent pH response enable the visual assessment of meat freshness. In this investigation, chitosan was initially employed as a colorimetric enhancer, leading to the development of a pH-sensitive indicator film that was enhanced in colorimetry. The characteristics of this indicator film were thoroughly analyzed, and the mechanism responsible for the increased sensitivity of anthocyanin within the chitosan matrix, as indicated by the color response, was elucidated. The recrystallization of chitosan impeded the hydration of AH^+^ as the pH increased from 6.0 to 8.0, leading to distinct color changes. Moreover, the application of this indicator film was extended to the monitoring of mutton meat freshness. It facilitated the differentiation of mutton meat into three distinct stages, namely, fresh, sub-fresh, and spoiled, based on alterations in color. Additionally, a robust positive correlation was established between the color difference value of the indicator film and the total volatile basic nitrogen and bacterial count of the mutton meat, enabling quantitative analysis. The present study, therefore, demonstrated a novel function of chitosan, i.e., the enhancement of the color of anthocyanin, which could be useful in designing and fabricating indicator films with a high color response.

## 1. Introduction

Stored meat could become contaminated with microorganisms, leading to the production of volatile biogenic amines and protein oxidation during meat storage, which ultimately causes pH changes in the internal environment of the packaging system [1]. An intelligent pH response indicator film is an easy-to-use device for the real-time monitoring of meat freshness during transit from the farm to the table [2]. These films are composed of two elemental parts: a pH-sensitive indicator that changes color with varying pH and the film-forming substrate onto which the indicator is immobilized [3].

Natural dyes have almost replaced chemical dyes as the preferred ideal choice for use in pH-sensitive indicators, as people across the globe are becoming increasingly aware of concerns related to food safety and environmental protection [4]. Anthocyanins extracted from blueberry [5], mulberry [6], purple sweet potato [7], eggplant [8], red cabbage [9], etc., have been used as biobased pH-sensitive dyes for monitoring the freshness of food in several previous studies. Natural biopolymers such as chitosan, gelatin, and carboxymethylcellulose have garnered significant attention in the development of indicator films due to their biocompatibility and nontoxic nature. Nevertheless, most anthocyanin indicator films tend to exhibit subpar color sensitivity. Two methods have been documented to enhance the color response sensitivity of anthocyanin indicator films. One approach involves the inclusion of a color enhancer. Bao et al. [10] introduced a pioneering method to enhance the pH sensitivity and colorimetry of anthocyanin indicator films through chondroitin sulfate copigmentation. Chondroitin sulfate forms a complex with anthocyanin, safeguarding flavonoid cations from nucleophilic water-based attacks, which results in more vivid and intense colors compared to anthocyanin alone. The second approach entails the addition of another natural pigment. Chen et al. [11] devised a novel pH-sensitive film containing both curcumin and anthocyanin. The incorporation of curcumin broadens the spectrum of color variation, thereby enhancing the sensitivity of anthocyanin indicator films. Intriguingly, our research revealed that when blueberry anthocyanin was immobilized onto a chitosan matrix, the pH-induced color changes exhibited increased sensitivity compared to the same changes observed in the blueberry anthocyanin solution. As chitosan has been commonly used as a film-forming substrate in intelligent food packaging systems, we are able to craft a pH-sensitive anthocyanin indicator film utilizing chitosan as both the film-forming substrate and colorimetry enhancer. This approach offers a dual advantage: enhancing pH response sensitivity without the need for additional substances and streamlining the fabrication process of highly pH-sensitive indicator films. We conducted a comparative investigation with two other conventional film-forming substrates, namely, gelatin and carboxymethylcellulose, to assess their potential to improve the sensitivity of anthocyanin. The results (Figure S1) demonstrate that only the chitosan-anthocyanin composite film exhibited outstanding pH response sensitivity, outperforming the gelatin-anthocyanin film and carboxymethylcellulose-anthocyanin film. Chitosan has been used successfully as an anthocyanin carrier in intelligent food packaging systems [12,13,14]. Nevertheless, the majority of investigations in this domain have predominantly centered on anthocyanin preparation and the physicochemical attributes of indicator films. In contrast, the influence of chitosan on the pH reactivity of anthocyanin and the mechanisms governing the interaction between chitosan and anthocyanin remain largely unexplored.

In this context, an intelligent indicator film with high pH sensitivity and excellent responsiveness was developed in the present study using chitosan as the colorimetry enhancer and film-forming substrate. The pH sensitivity of blueberry anthocyanin was observed to improve when it was embedded into a chitosan substrate. Therefore, as the next step of the study, the mechanism underlying the color response-based sensitivity enhancement of anthocyanin with the use of the chitosan matrix was explored. In addition, the structure of the indicator film was characterized, and the color responses and storage stability of the developed film were evaluated. Finally, the developed chitosan-blueberry anthocyanin indicator film was applied to the real-time monitoring of mutton meat freshness. The outcomes of this current investigation are anticipated to unveil a new role for chitosan and present a straightforward approach for augmenting the color sensitivity of anthocyanin indicator films devoid of any supplementary additives. These findings may offer valuable insights for the design and production of anthocyanin indicator films with increased color responsiveness.

## 2. Materials and Methods

### 2.1. Materials

Cyanidin cation powder (purity: 25%; extracted from blueberry, BA) and Chitosan (deacetylated degree: ≥95%; viscosity: 100–200 mPa·s) were purchased from Shanghai Aladdin Biochemical Technology Co., Ltd., (Shanghai, China). Mutton meat was bought from Quanfu Life Square (Jinan, China). All remaining reagents used in the study were of analytical grade.

### 2.2. Preparation of the Chitosan–BA Indicator Films

First, a 1% chitosan solution was prepared by dissolving chitosan in 0.05% HCl solution. Next, a stock solution of BA (10 g/L) was prepared by dissolving BA in 0.01% HCl solution and added to the above 1% chitosan solution in different amounts to obtain the final film-forming solutions containing 0, 0.1, 0.3, and 0.5 g/L concentrations of BA. Each film-forming solution was stirred gently for 30 min to obtain a homogenous solution. Afterward, a 60 mL film-forming homogenous solution was cast on a clean acrylic mold (100 mm × 100 mm × 10 mm), which was maintained in a drying oven at 40 °C for 24 h to allow for the formation of the indicator film. The obtained indicator films were designated according to the amount of BA added, as C-BA-0 (without BA), C-BA-1 (with 0.1 g/L BA), C-BA-3 (with 0.3 g/L BA), and C-BA-5 (with 0.5 g/L BA).

### 2.3. Color Measurement

The color changes in the C-BA-1, C-BA-3, and C-BA-5 indicator films were recorded using a camera (Canon EOS 70D (W), Canon, Tokyo, Japan). The L*, a*, and b* values of the indicator films were measured using a colorimeter (CR-400, Konica Minolta, Tokyo, Japan). The RGB color models of the indicator films were analyzed using MATLAB R2014a. The color difference value (ΔE) and the response sensitivity (S_RGB_) of the indicator films were calculated using Equation (1) and Equation (2) [15], respectively, which are provided below.

(1) $$\Delta E=\sqrt{{\left(L^{*}-L_{0}^{*}\right)}^{2}+{\left(a^{*}-a_{0}^{*}\right)}^{2}+{\left(b^{*}-b_{0}^{*}\right)}^{2}}$$

(2) $$S_{\mathrm{RGB}}=\frac{\left(R-R_{0}\right)+\left(G-G_{0}\right)+(B-B_{0})}{R+G+B}\times 100$$

In the above equations, L denotes lightness, a denotes the redness-greenness, and b denotes the yellowness-blueness of the indicator films; R denotes red, G denotes green, and B denotes blue; L_0_*, a_0_*, b_0_*, R_0_, G_0_, and B_0_ are the values of the control.

### 2.4. pH Sensitivity

The samples of each indicator film (10 mm × 10 mm) were immersed in buffer solutions with pH 6.0, 6.5, 7.0, 7.5, or 8.0 for 30 s. Afterward, the L, a, and b values of each sample were measured using a colorimeter, and a camera was employed to record sample color changes. The ΔE values were then calculated using Equation (1). L_0_, a_0_, and b_0_ were used as the color parameters of the unsoaked original film.

### 2.5. Ammonia Sensitivity

The indicator film was fixed on the mouth of a conical flask (100 mL) containing 10 mL of ammonia water (20%, *v*/*v*). Afterward, the color changes in the indicator film were recorded using the colorimeter and the camera at intervals of 1 min, for a total of 6 min [16].

### 2.6. Characterisation of the Prepared Indicator Film

#### 2.6.1. Thickness

The thickness of the indicator films were measured with a Mitutoyo digital micrometer (precision is 0.001 mm, Tester Sangyo Co., Ltd., Saitama, Japan) at 5 random locations of each film.

#### 2.6.2. Moisture Content

The moisture content of the indicator films was determined by calculating the mass loss for each sample. Each indicator film sample was dried at 105 °C until a constant weight was reached.

(3) $$\mathrm{Moisture}\;\mathrm{content}\;(\%)=\frac{W_{0}-W_{1}}{W_{0}}\times 100\%$$

where W_0_ and W_1_ denote the original and final weight of the indicator film sample, respectively.

#### 2.6.3. Water Solubility

The water solubility of the indicator films was measured according to wang [13] with little modification. The indicator film sample (50 mm × 50 mm) was immersed in 50 mM sodium phosphate buffer (pH 7.0) at 25 °C for 24 h. The insoluble matter was then separated through centrifugation at 8000 rpm for 15 min, followed by drying at 105 °C until a constant weight was reached. The water solubility was calculated using the following expression:

(4) $$\mathrm{Water}\;\mathrm{solubility}\;(\%)=\frac{W_{a}\times (1-M)-W_{b}}{W_{a}\times (1-M)}\times 100\%$$

where W_a_ and W_b_ denote the original and final weight of the indicator film sample, respectively, and M denotes the moisture content of the indicator film sample.

#### 2.6.4. Water Vapor Barrier Property

The indicator film was tightly covered on the mouth of a test cup containing anhydrous silica gels. The cup was weighted and kept in the desiccator with 100% relative humidity at 20 °C. The weight change of the cup was recorded at intervals of 4 h for a total of 3 days [13]. The water vapor permeability (WVP) was calculated according to the following expression:

(5) $$\mathrm{WVP}=\frac{W\times h}{t\times S\times ∆P}$$

where, W denotes the gain in cup weight (g), h denotes the thickness (m) of the indicator film sample, t denotes time (s) for the gain in cup weight, S denotes the area of the indicator film sample for permeation (m^2^), and ΔP denotes partial vapor pressure.

#### 2.6.5. Mechanical Properties

The tensile tests were conducted by a texture analyzer (TA series, Stable Micro System Co., Ltd., Godalming, UK). The samples (80 mm × 15 mm) were tested at a gauge length of 40 mm and strain rate of 5 mm min^−1^. The tensile strength and elongation were recorded as the stress and increased length ratio at sample breakage.

#### 2.6.6. Color Stability

The L*, a*, and b* values of the indicator films at 4 °C were measured at intervals of 2 days for a total of 14 days. The ΔE values during storage were calculated using Equation (1) and the original film as a reference.

#### 2.6.7. Fourier Transformation Infrared Spectroscopy (FT-IR) Analysis

The FT-IR spectra of the indicator films were recorded using an FT-IR spectrometer (Nicolet iS 20, Thermo Scientific, Waltham, MA, USA) operated at the attenuated total reflection (ATR) mode. The transmittance (%) data were collected within the wavenumber range of 600 cm^−1^ to 4000 cm^−1^ and then plotted as a function of wavenumber (cm^−1^). OMNIC 8.2 software (Thermo Scientific, Waltham, MA, USA) was employed to analyze the recorded spectra.

#### 2.6.8. X-ray Diffraction (XRD) Analysis

The XRD patterns of the indicator films were recorded using an X-ray diffractometer (Ultima IV, Rigaku, Tokyo, Japan) with Cu Ka set at 40 kV and 40 mA. The intensity data were collected for the 2θ values ranging from 5° to 60° with a step-up of 2°. The resultant images were exported to the OriginPro 2018C software (© OriginLab Corporation, Northampton, MA, USA) for analysis.

### 2.7. Application of the Prepared Indicator Films for Monitoring Mutton Freshness

#### 2.7.1. Fresh Mutton Spoilage Trial

The mutton meat was cut into 25 pieces (each weighing 100.0 ± 5.0 g) and placed into 25 sterilized plastic packaging boxes (250 cm^3^). The indicator film was attached to the lid of each box without any direct contact with the mutton meat. One of the mutton samples was used to record the color changes of indicator film, and the others were randomly divided into 8 groups for spoilage trial. All the samples were stored at 4 °C for 7 days. The total volatile basic nitrogen (TVB-N) and total viable count (TVC) of the mutton meat samples were determined once every day using one group of samples. Meanwhile, the color parameters (L*, a*, and b*) of the indicator films were also recorded.

#### 2.7.2. TVB-N Analysis

The TVB-N content of each sample was measured according to the Chinese Standard [17] Each sample weighing 10 g was first homogenized and then dispersed and impregnated in distilled water (50 mL) for 30 min. Afterward, the sample was filtered, and 10 mL of the filtrate was mixed with 5 mL of MgO suspension (10 g/L) in a reaction chamber. The distillate was collected in a receiving flask containing 10 mL of a boric acid solution (20 g/L). Titration was performed using 0.01 M of the standard hydrochloric acid titration solution until the endpoint was reached. The TVB-N value was calculated in terms of the amount of standard hydrochloric acid consumed during titration and expressed as mg/100 g.

#### 2.7.3. TVC Analysis

The TVC of each sample was determined according to the Chinese Standard [18] using the plate counting method. Each sample weighing 25 g was first homogenized in 225 mL of sterilized normal saline, after which the 10^−1^–10^−4^ dilutions of the homogenate were subjected to microbial counting. The TVC value was expressed as log CFU (colony forming units) g^−1^.

### 2.8. Statistical Analysis

The results were expressed as the mean value ± standard deviation of at least 3 experiments. All result data were analyzed statistically through ANOVA using SPSS version 22.0 (SPSS Inc., Chicago, IL, USA). A *p*-value of <0.05 was considered statistically significant.

## 3. Results and Discussion

### 3.1. Determination of the BA Content in the C-BA Indicator Films

The initial color appearance of indicator films is an important index used in evaluating their application [19]. As presented in Table 1, the prepared indicator films exhibited different colors with the addition of different concentrations of anthocyanin ranging from 0.1 g/L to 0.5 g/L. Correspondingly, the color parameters for these indicator films were also significantly different (*p* < 0.05). These results indicated that the BA content could be related to the color responses of the indicator films.

#### 3.1.1. Ammonia Sensitivity

**Figure 1:**
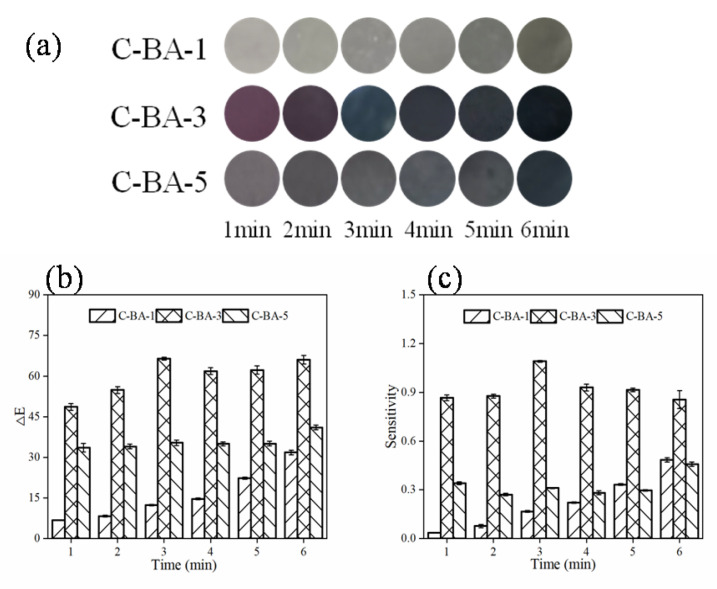
The color change (**a**), ΔE value (**b**), and sensitivity (**c**) of chitosan-anthocyanin film with 0.1 g/L (C-BA-1), 0.3 g/L (C-BA-3) and 0.5 g/L (C-BA-5) blueberry anthocyanin mass concentration to volatile ammonia.

The response of the prepared indicator films to volatile nitrogen compounds produced during meat spoilage was estimated by determining the reaction sensitivity of these indicator films to ammonia vapor. The results (Figure 1) revealed that the C-BA-3 film exhibited more apparent color changes than the C-BA-1 and C-BA-5 films, suggesting that the film color change was linked to the inclusion of BA [14]. The ΔE value and the S_RGB_ score increased during the first 3 min, which suggested that the C-BA-3 film exhibited the most rapid response to ammonia vapor among all indicator films. The gradient color changes and the rapid response of the C-BA-3 film to ammonia indicated its potential for application in the monitoring of meat freshness.

#### 3.1.2. pH Sensitivity

**Figure 2:**
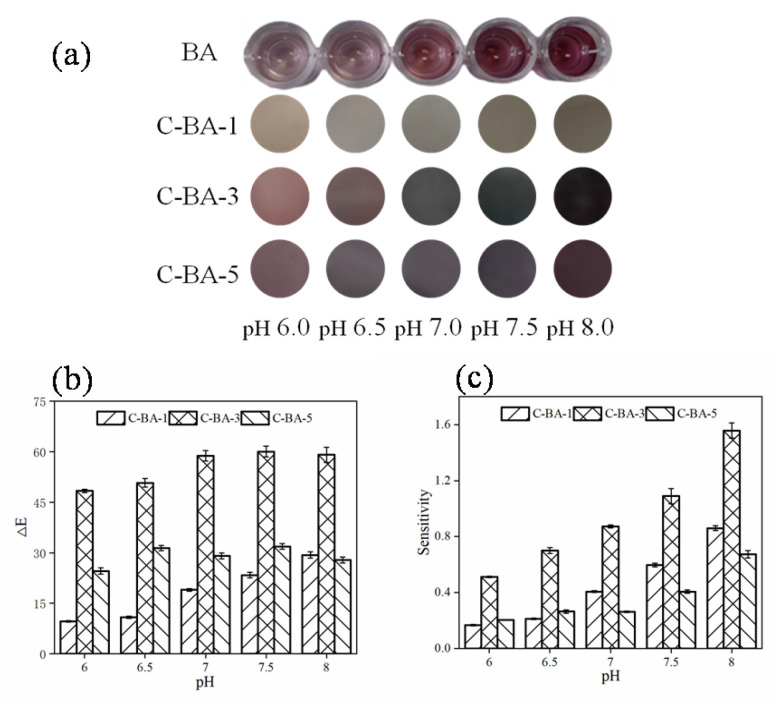
The color change (**a**), ΔE value (**b**), and sensitivity (**c**) of anthocyanin (BA) and chitosan-anthocyanin composited film with 0.1 g/L (C-BA-1), 0.3 g/L (C-BA-3) and 0.5 g/L (C-BA-5) blueberry anthocyanin mass concentration at pH 6.0–8.0.

The pH of most animal products during storage reportedly ranges from 6.0 to 8.0 [10]. Therefore, the color responses of the indicator films prepared in the present study were evaluated in buffer solutions with pH = 6.0–8.0. The results, presented in Figure 2, revealed that the pH-induced color changes in the indicator films were different from those observed in the BA solution, which was attributable to copigmentation with the chitosan molecule [20,21]. The C-BA-3 indicator film exhibited more evident color changes compared to the C-BA-1 and C-BA-5 films, and the color varied from red to red-bluish violet as the pH changed from 6.0 to 6.5. At pH 7.0, the color appeared grayish-blue, which then changed to blue-gray (pH 7.5–8.0). All color changes were distinguishable by the naked eye. The high color responsiveness and pH sensitivity of the C-BA-3 film suggested its great potential for application in meat freshness monitoring.

According to the above results, the C-BA-3 indicator film was selected for subsequent analysis because of its outstanding performance in the colorimetric response to ammonia and pH range analysis.

### 3.2. Characterization of the C-BA-3 Indicator Film

#### 3.2.1. Thickness, Moisture Content, and Water VAPOR Permeability Analysis

As shown in Table 2, the thickness, moisture content, and water vapor permeability of the C-BA-3 indicator film were not very different from that of the C-BA-0 film. The thickness of the C-BA-3 indicator film was similar to that of the C-BA-0 film, indicating that the addition of BA could make chitosan film become compact as it was well distributed in the chitosan matrix. The incorporation of BA could establish intermolecular interactions between anthocyanin and hydroxyl/amino groups in chitosan chains, inhibiting water molecule-chitosan interactions, leading to a little lower moisture content in C-BA-3 indicator film compared with C-BA-0 film [13,22]. The different WVP between C-BA-3 indicator film and C-BA-0 film might be attributed to the compact structure of C-BA-3; BA acts as a bridge among different chitosan chains, reducing the permeability of water vapor.

#### 3.2.2. Mechanical Property Analysis

The tensile strength and elongation at break represent the mechanical resistance and flexibility of the film, respectively. As can be seen from Table 2, the tensile strength of chitosan film increased from 16.20 Mpa to 19.03 Mpa, and the elongation at break of chitosan film decreased from 34.83% to 25.37% with the addition of BA. The relatively high mechanical resistance might be due to the compact structure in the C-BA-3 indicator film. At the same time, the relatively low flexibility might be due to the fewer water molecule interactions within the C-BA-3 indicator film [23].

#### 3.2.3. FT-IR Analysis

**Figure 3:**
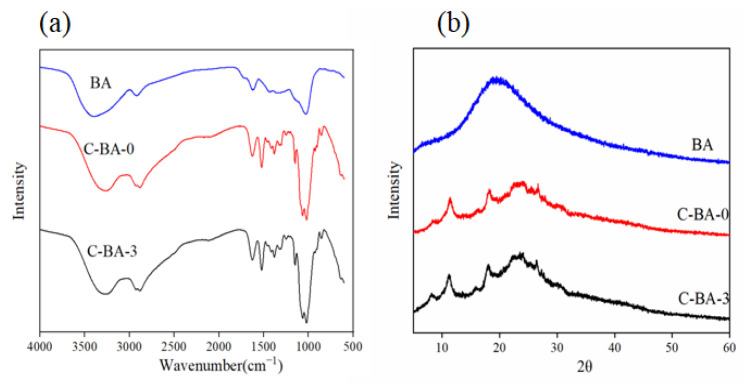
FT-IR spectra (**a**) and XRD patterns (**b**) of BA, C-BA-0 film, and C-BA-3 indicator film.

The characterization of the functional groups in the structure of the prepared film and the interactions among the film components were evaluated for the C-BA-3 indicator film using FT-IR [24]. The results, presented in Figure 3a, revealed the presence of a wide BA band at 3395 cm^−1^, which was attributed to O–H stretching. Two bands appeared at approximately 1634 cm^−1^ and 1028 cm^−1^, which were attributed to C=C stretching and the C–H deformation of aromatic rings, respectively. The spectra of the C-BA-3 indicator film were highly similar to those of the C-BA-0 film, with both having a broad band at around 3280 cm^−1^ (attributed to O–H and N–H stretching, respectively) [25] and two additional bands at approximately 2920 cm^−1^ (corresponding to C–H stretching) and 1406 cm^−1^ (C–H bending) [4]. The bands that appeared at 1633 and 1551 cm^−1^ were assigned to C=O stretching and N–H bending, respectively [7]. The bands appearing at 1151 cm^−1^ and 1026 cm^−1^ corresponded to C–O–C stretching and C–O stretching, respectively, which were related to the saccharide structure [26]. These results suggested that the low content of BA in the C-BA-3 indicator film exerted little effect on the interaction with chitosan molecules.

#### 3.2.4. XRD Analysis

The crystalline nature of the prepared films was analyzed using XRD. As depicted in Figure 3b, the XRD patterns of the C-BA-3 indicator film did not change significantly after the addition of BA. Each film exhibited five diffraction peaks at 2θ = 8.4°, 11.3°, 16.0°, 18.0°, and 23.0°, which were attributed to the semi-crystalline structure of chitosan [7]. In comparison to the C-BA-0 film, the C-BA-3 indicator film exhibited a higher diffraction peak intensity at 2θ = 8.4° and 16.0°, suggesting that the crystalline degree of the C-BA-0 film was slightly increased after the incorporation of BA. Yong [8] reported that the crystallinity of an anthocyanin-rich film was attributable to the content and composition of the incorporated anthocyanin. Therefore, the addition of BA could have increased the crystallinity of the C-BA-3 indicator film to a certain extent.

#### 3.2.5. Possible Mechanism Underlying the Color Response Sensitivity Enhancement of Anthocyanin in the C-BA-3 Indicator Film

To investigate the effect of chitosan on the pH responses of anthocyanin and the interaction mechanism underlying the effect of the C-BA-3 indicator film, the XRD patterns and the FT-IR spectra of the C-BA-3 indicator film recorded at the key color-turning points (pH 6.0, pH 7.0, and pH 8.0) were examined. In the FTIR spectra, no new bands appeared as the pH varied from 6.0 to 8.0, suggesting no covalent bond formation in the C-BA-3 indicator film during this process. In the XRD patterns, the diffraction peak intensity was significantly increased as the pH increased from 6.0 to 8.0, indicating that the semi-crystalline structure of chitosan in C-BA-3 indicator film was related to the degree of alkalinity. Moreover, the increase in the intensity at 2θ = 11.3°, which was the hydrated crystalline peak, indicated the formation of new hydrogen bonds between chitosan and water molecules in the C-BA-3 indicator film [27]. This implied that the water molecules were more inclined to bind to chitosan.

**Figure 4:**
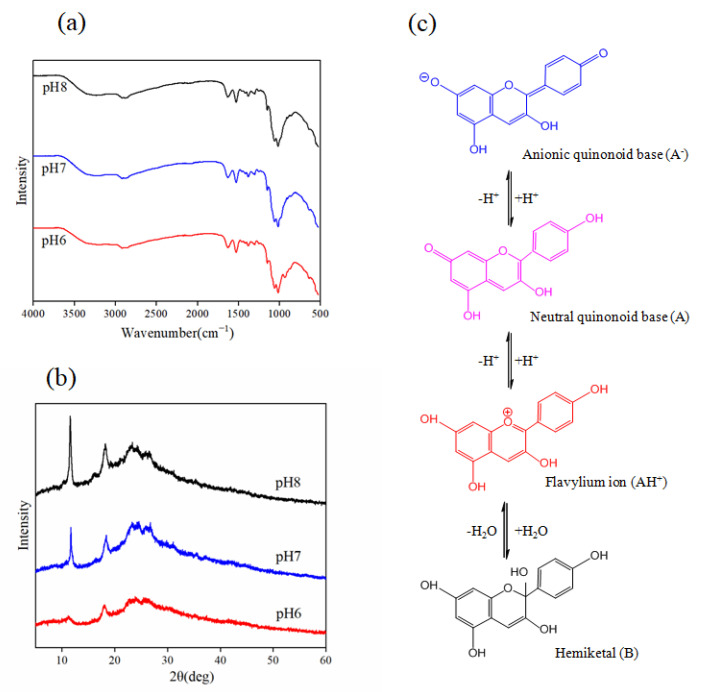
FT-IR spectra (**a**) and XRD patterns (**b**) of C-BA-3 indicator film at pH 6.0, pH 7.0, pH 8.0 and structural transformations of BA in acidic to neutral conditions (**c**).

According to previous research [28], anthocyanin is present mainly in the form of flavylium cations (AH^+^) at pH < 2. Above pH 2, the red flavylium ion (AH^+^) first loses a proton due to the mildly acidic conditions, generating the purple quinonoid base (A), and then another proton is lost, which generates the blue anionic quinonoid base (A^−^) at neutrality. Meanwhile, hydration of the AH^+^ ions also occurs, which generates the colorless hemiketal (B) in acidic-to-neutral conditions (Figure 4). The color response is determined by the forms of anthocyanin present at a particular time under given pH values. As the pH increased from 6.0 to 8.0, water molecules were more inclined to bind to chitosan, which impeded the hydration of AH^+^, thereby reducing the formation of colorless hemiketal (B) and improving the colorimetric response sensitivity of the C-BA-3 indicator film. These results demonstrated that the colorimetric sensitivity of the C-BA-3 indicator film was related to the spatial conformation of the chitosan molecules.

#### 3.2.6. Water Solubility

**Figure 5:**
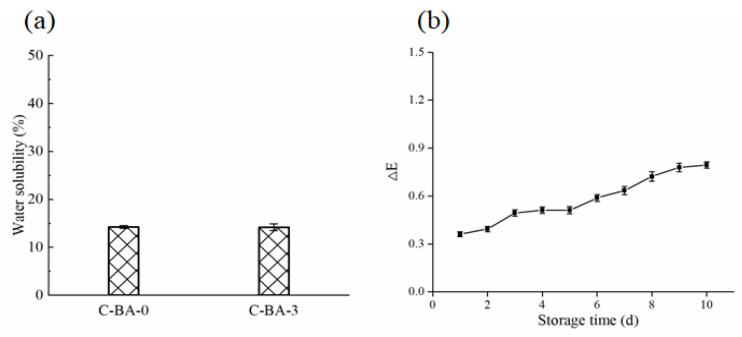
Water solubility of C-BA-0 and C-BA-3 indicator film (**a**) and color stability of C-BA-3 indicator film at 4 °C within 10 days (**b**).

An efficient indicator film should exhibit good water resistance and maintain integrity during meat storage [29]. Therefore, the water solubility of the C-BA-3 indicator film was determined. As presented in Figure 5a, the water solubility of the C-BA-3 indicator film was similar to that of the C-BA-0 film, with 14.2% of the film dissolving in water after 24 h. The water solubility might be due to the presence of hydrophilic groups in chitosan [30]. The hydrophobic groups in the C-BA-3 indicator film would be helpful for its application in intelligent food packaging systems.

#### 3.2.7. Color Stability of the C-BA-3 Indicator Film

According to previous research [3], temperature impacts the stability of anthocyanin. Therefore, considering the actual storage environment of fresh meat, the change in the ΔE value of the C-BA-3 indicator film was evaluated for storage at 4 °C for 10 days. As shown in Figure 5b, the C-BA-3 indicator film exhibited excellent color stability, with ΔE less than 0.79 after 10 days. This outstanding performance demonstrated its great potential for application in intelligent packaging systems.

### 3.3. Application of the C-BA-3 Indicator Film in Monitoring Mutton Meat Freshness

**Figure 6:**
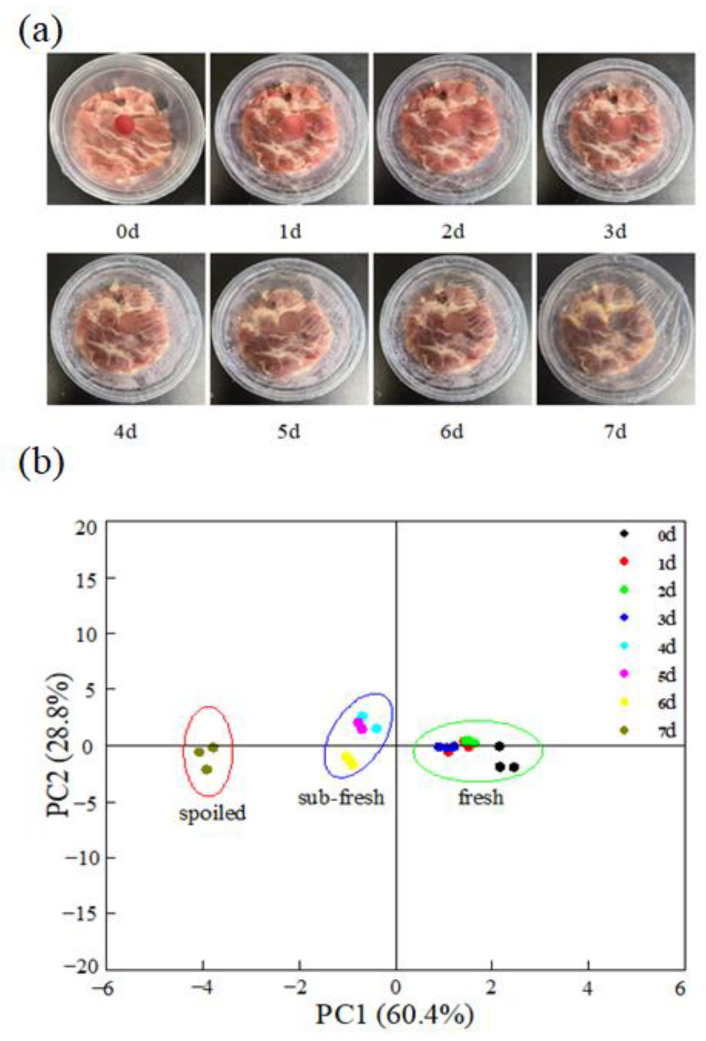
Color change of C-BA-3 indicator film within 7 days at 4 °C (**a**) and PCA plots of freshness discrimination by color indicators (**b**).

Fresh mutton meat samples were stored at 4 °C for 7 days, after which their TVB-N and TVC values were measured. In addition, the color changes in the C-BA-3 indicator film were recorded during the storage period (Figure 6a). According to GB 9961–2008 [31], a TVB-N level of 15 mg/100 g or above is a condition for rejecting the mutton meat sample. As presented in Table 3, the TVB-N value of fresh mutton meat was 15.53 mg/100 g on Day 7, which suggested that the mutton meat had been spoilt completely. Meanwhile, the TVC value of this mutton meat was 6.94 [lg (CFU/g)], and the corresponding indicator film color turned grayish-blue, indicating the critical point of spoilage of fresh mutton meat. In the mutton storage period, the indicator film exhibited a significant color change, from red to blue to violet to grayish-blue, and all of these colors were distinguishable by the naked eye.

PCA based on color indicators (L*, a*, b*, R, G, B) was performed to test whether the color difference could adequately distinguish the freshness levels of the mouton meat. As shown in Figure 6b, the fresh states of the mouton were categorized into “fresh” (0–3 d), “sub-fresh” (4–6 d), and “spoiled” (7 d) based on the color variations observed in C-BA-3 film, which is inconsistent with the results of hierarchical cluster analysis and the Fisher discriminant model based on traditional quality indices and sensory scores [32]. Furthermore, the ΔE of the C-BA-3 indicator film was observed to have a high positive correlation with the TVB-N and TVC values of mutton meat (Table 4). A close relationship between the TVB-N and TVC values of mutton meat was also observed. Collectively, these results suggested that the C-BA-3 indicator film has great potential for application in the field of mutton quality assessment.

## 4. Conclusions

A chitosan/blueberry anthocyanin indicator film (C-BA-3) with excellent pH sensitivity and color responses was developed. The C-BA-3 indicator film exhibits high responsiveness in the pH range of 6.0–8.0, and its color response sensitivity is related to the structural transformation of chitosan molecules. When the C-BA-3 indicator film was used for the real-time monitoring of mutton freshness at 4 °C, the freshness was differentiated into three states via distinct color changes of the indicator film. Moreover, the color changes in the film demonstrated a high correlation with the TVB-N and TVC values of the mutton meat during storage. Therefore, the fabricated C-BA-3 indicator film has the potential for application in meat freshness monitoring and intelligent packaging systems. The present study contributes to the current and future efforts for designing and fabricating high-color-response anthocyanin indicator films.

## Figures and Tables

**Table 1:** Color and color parameters of indicator films.

Film	C-BA-1	C-BA-3	C-BA-5
photograph	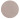	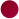	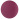
L*	65.68 ± 0.94 ^c^	41.46 ± 0.11 ^a^	49.43 ± 0.24 ^b^
a*	6.17 ± 0.25 ^a^	35.53 ± 0.05 ^c^	19.88 ± 0.18 ^b^
b*	0.63 ± 0.028 ^b^	5.23 ± 0.22 ^c^	−3.96 ± 0.02 ^a^

Chitosan-anthocyanin composited films with 0.1 g/L (C-BA-1), 0.3 g/L (C-BA-3), and 0.5 g/L (C-BA-5) blueberry anthocyanin mass concentration. Letters with different superscripts in the same column indicate a significant difference (*p* < 0.05).

**Table 2:** Thickness, moisture content, water vapor permeability, and mechanical properties of C-BA-0 and C-BA-3 film.

Film	Thickness	Moisture Content	Water Vapor Permeability	Tensile Strength	Elongation at Break
	μm	%	10^−11^ g m^−1^ s ^−1^ Pa^−1^	Mpa	%
C-BA-0	100.00 ± 1.25	28.59 ± 2.16	16.29 ± 0.23	16.20 ± 0.56	34.83 ± 1.00
C-BA-3	100.10 ± 1.13	27.67 ± 2.55	15.97 ± 0.51	19.03 ± 0.25	25.37 ± 1.12

**Table 3:** Changes in TVB-N, TVC of stored fresh mutton meat, and ΔE values of C-BA-3 indicator film within 7 days at 4 °C.

Storage Time (Day)	TVB-N (mg/100 g)	TVC (lg (CFU/g))	ΔE
0	8.82 ± 0.18	3.60 ± 0.01	—
1	8.91 ± 0.30	3.68 ± 0.01	10.72 ± 0.31
2	10.24 ± 0.62	3.98 ± 0.02	19.72 ± 0.42
3	10.26 ± 0.58	5.04 ± 0.08	20.69 ± 0.23
4	11.16 ± 0.36	5.48 ± 0.02	29.22 ± 0.34
5	12.09 ± 0.10	5.62 ± 0.02	32.71 ± 0.47
6	14.34 ± 0.70	5.70 ± 0.02	33.82 ± 0.5
7	15.53 ± 0.37	6.94 ± 0.02	51.79 ± 0.11

**Table 4:** The correlation coefficient between TVB-N, TVC, and ΔE.

	TVB-N	TVC	ΔE
TVB-N	1		
TVC	0.713 **	1	
ΔE	0.921 **	0.731 **	1

** correlation is significant at the 0.01 level (2-tailed).

## Data Availability

All data generated and analyzed during this study are included in this article.

## Supplementary Materials

The following supporting information can be downloaded at: https://www.mdpi.com/article/10.3390/polym16060849/s1, Figure S1: The color change (a), ΔE value (b) and sensitivity (c) of CMC-BA film, Chitosan-BA film and Gelatin-BA film at pH 6.0–8.0.
