# Supplementary material for: Chitosan-Enhanced pH-Sensitive Anthocyanin Indicator Film for the Accurate Monitoring of Mutton Freshness
Source: Polymers (Basel). 2024 Mar 20;16(6):849. doi: 10.3390/polym16060849 (PMC10974092; doi:10.3390/polym16060849)
Supplement: Supplementary file 1 [file polymers-16-00849-s001.zip › polymers-2885324-supplementary.pdf]

Chitosan-Enhanced pH-Sensitive Anthocyanin Indicator Film for the Accurate Monitoring of Mutton Freshness

(Supporting Information)

Yanli Ma [1], Lei Wen [1,2], Yaobo Liu [1], Pengfei Du [1], Peng Hu [1], Jianfang Cao [1] and Weiting Wang [1,*]

[1] Shandong Provincial Key Laboratory of Agro-Products Processing Technology, Key Laboratory of Novel Food Resources Processing, Ministry of Agriculture, Institute of Food & Nutrition Science and Technology, Shandong Academy of Agricultural Sciences, Jinan 250100, China
[2] College of Life Sciences, Yantai University, Yantai 264005, China
* Correspondence: wangweiting0619@163.com; Tel.: +86-0531-66655263

S1 Materials and Methods

S1.1. Materials

Anthocyanin (25%; extracted from blueberry, BA) and chitosan (deacetylated degree: ≥95%; viscosity: 100–200 MPa.s), Gelatin (250g Bloom) and Sodium Carboxymethylcellulose (CMC) were purchased from Shanghai Aladdin Biochemical Technology Co. Ltd. (Shanghai, China). All remaining reagents used in the study were of analytical grade.

S1.2. Preparation of BA indicator films

First, 1% chitosan solution was prepared by dissolving chitosan in 0.15% HCl solution, 1% CMC solution and 1% gelatin solution were prepared by dissolving CMC and gelatin in deionized water, respectively. Next, a stock solution of BA was prepared and added to the above solution to obtain the final film-forming solutions

containing 0.3 mg/mL concentrations of BA. Each film-forming solution was stirred gently for 30 min to obtain a homogenous solution. Afterward, each film-forming homogenous solution was cast on a clean acrylic mold, which was maintained in a drying oven at 40 °C for 24 h to allow for the formation of the indicator films. The obtained indicator films were designated according to the film-forming substrate, as CMC-BA, Chitosan-BA and Gelatin-BA.

S1.3. pH sensitivity

The samples of each indicator film (10 mm × 10 mm) were immersed in buffer solutions with pH 6.0, 6.5, 7.0, 7.5, or 8.0 for 30 s. Afterward, the L, a, and b values of each sample were measured using a colorimeter, and a camera was employed to record sample color changes. The RGB colour models of the indicator films were analysed using MATLAB R2014a. The color difference value (ΔE) and the response sensitivity (S_{RGB}) of the indicator films were calculated using Eq. (1) and Eq. (2), respectively, which are provided below.

$$\Delta E = \sqrt{(L - L_0)^2 + (a - a_0)^2 + (b - b_0)^2} \quad \text{(S1)}$$

$$S_{RGB} = \frac{|R-R_0| + |G-G_0| + |B-B_0|}{R+G+B} \times 100 \quad \text{(S2)}$$

In the above equations, L denotes lightness, a denotes redness-greenness, and b denotes yellowness-blueness of the indicator films; L_0, a_0 b_0, R_0 (red), G_0 (green), and B_0 (blue) are the values of the control.

S2 Results

The Chitosan-BA indicator film presented obvious color changes, high color responsiveness and pH sensitivity compared with CMC-BA film and Gelatin-BA film,

suggesting that chitosan might be a colorimetry enhancer of BA.

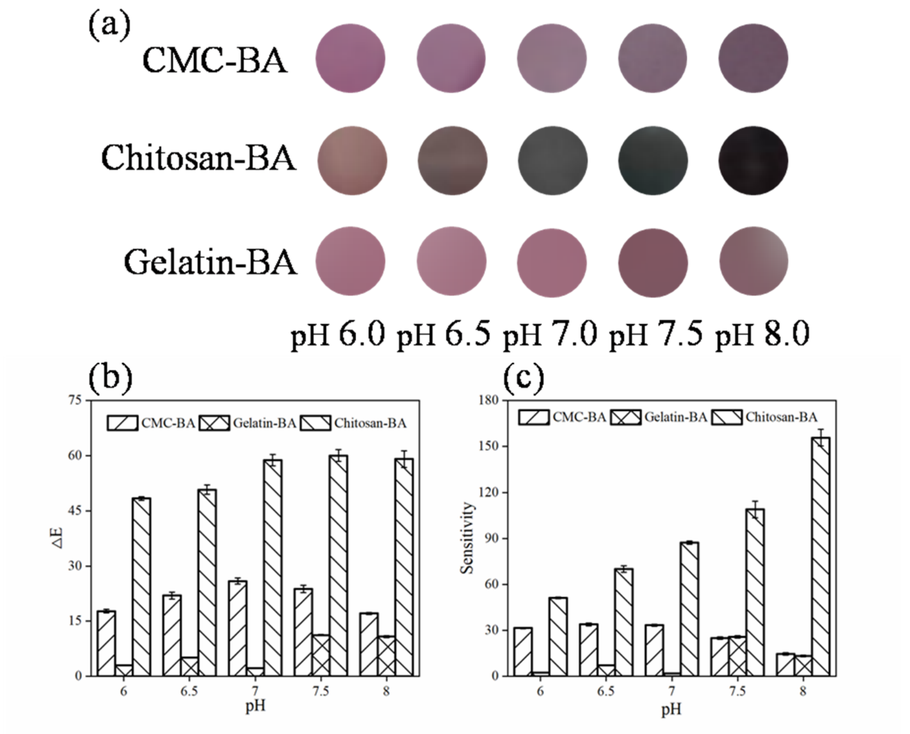

Figure S1. The color change (a), ΔE value (b) and sensitivity (c) of CMC-BA film, Chitosan-BA film and Gelatin-BA film at pH 6.0-8.0.
